# Targeting non-coding RNA H19: A potential therapeutic approach in pulmonary diseases

**DOI:** 10.3389/fphar.2022.978151

**Published:** 2022-09-16

**Authors:** Jinghui Xie, Yuedi Hu, Dengdi Sun, Changan Liu, Zegeng Li, Jie Zhu

**Affiliations:** ^1^ College of Integrated Chinese and Western Medicine, Anhui University of Chinese Medicine, Hefei, Anhui, China; ^2^ The Key Laboratory of Intelligent Computing and Signal Processing (ICSP), Ministry of Education, School of Artificial Intelligence, Anhui University, Hefei, China; ^3^ Institute of Traditional Chinese Medicine Prevention and Control on Respiratory Disease, Anhui Academy of Chinese Medicine, Hefei, Anhui, China; ^4^ Department of Respiratory Medicine, First Affiliated Hospital of Anhui University of Traditional Chinese Medicine, Hefei, Anhui, China; ^5^ Institutes of Integrative Medicine, Fudan University, Shanghai, China

**Keywords:** drugs, lung diseases, mechanisms, competition, long non-coding RNA H19

## Abstract

Non-coding RNA is still one of the most popular fields in biology research. In recent years, people paid more attention to the roles of H19 in lung diseases, which expressed abnormally in various pathological process. Therefore, this review focus on the regulatory role of H19 in asthma, pulmonary arterial hypertension (PAH), idiopathic pulmonary fibrosis (IPF), lung injury, pneumonia, lung cancer, etc. And the potential therapeutic agents and molecular treatments of H19 are collected. The aim is to demonstrate its underlying mechanism in pulmonary diseases and to guide the basic research targeting H19 into clinical drug translation.

## 1 Introduction

The central dogma of genetic inheritance states that DNA is transcribed into RNA, which is then translated into protein. However, protein-coding RNAs make up only a proportion of total RNA, leaving more non-coding RNAs. Non-coding RNAs are now known to have many regulatory functions concerning gene expression, and interactions with DNA, RNA, and protein have all been reported (Hangauer et al., 2013). Non-coding RNAs act as inducers, mediators, guides, or signal molecules, influencing many biological processes, including cell proliferation and differentiation (Wang et al., 2021a). For example, with respect to pulmonary dysfunction, the lncRNA TUG1 has been found to sponge miR-222-3p with the result that the expression of the genes encoding CELF1 and p53 are up-regulated (Li et al., 2021).

Non-coding RNAs are broadly divided into two classes based on their length, with long non-coding RNAs (lncRNA) having greater than 200 nucleotides and short non-coding RNAs, predominantly micro-endogenous RNAs having fewer than 200 nucleotides. LncRNAs, in particular, have been extensively studied and are known to have many functions (Li et al., 2019a).

One member of the lncRNA family, H19, is abundant and shows a high degree of conservation. Its involvement in cancer (Muller et al., 2019), diabetes (Li et al., 2020a), cardiovascular (Zhang et al., 2018), and cerebrovascular disease (Feng et al., 2021) has been reported. For example, H19 binds hnRNPA2B1 and induces the epithelial-mesenchymal transformation, and promotes metastasis and invasion of colorectal cancer cells (Zhang et al., 2020a).

In recent years, research on drugs targeting lncRNAs has gradually emerged, and reviews have discussed the progress and challenges encountered in the development of lncRNAs as potential biomarkers and molecular drugs (Winkle et al., 2021). Similarly, H19, a clinical marker with broad application prospects, is more and more concerned in lung diseases. The review aims to summarize the roles of H19 in pulmonary diseases to reveal the therapeutic target and the direction to improve treatment.

## 2 The origin of H19

H19 is a maternally imprinted gene located on the short arm of chromosome 11 at locus 11P15.5 (Ghafouri-Fard et al., 2020). It is a single-copy gene that shows a high degree of conservation. The 2.5 kb length consists of 5 exons and 4 introns and generates a mature 2.3 kb transcript lacking an open reading frame (Cai and Cullen, 2007). H19 is expressed at a high rate during development *in utero* but is down-regulated after birth, with heart and skeletal muscle being two of the few tissues that retain residual expression (Martinet et al., 2016). The expression may be detected predominantly in the cytosol, but H19 is also present in the nucleus (Nordin et al., 2014). Transcription is considered to result from the activity of RNA polymerase II and an antisense RNA molecule of H19, 91H, has also been detected, although its function remains unclear (Pachnis et al., 1988; Berteaux et al., 2008).

## 3 The regulatory roles of H19

**FIGURE 1:**
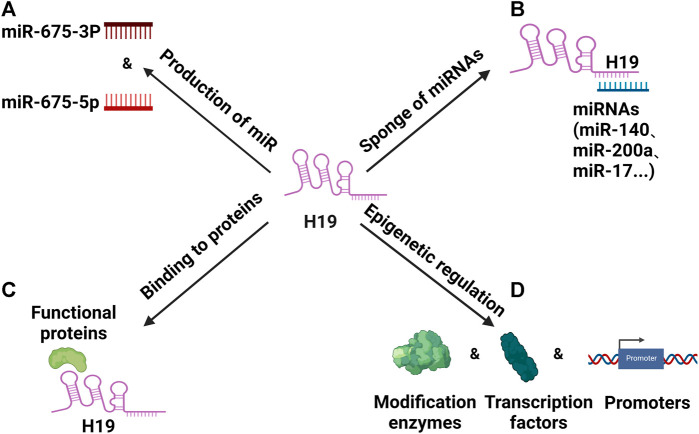
The regulatory roles of H19. **(A)** H19 can indirectly regulates downstream molecules by its spliceosome miR-675-3p or miR-675-5p. **(B)** H19 can regulates downstream molecules by acting as a “miRNA sponge”. **(C)** H19 can regulates downstream molecules by binding to functional proteins. **(D)** By epigenetic pathways, H19 can regulate modification enzymes, transcription factors and promoters to regulate downstream molecular expression.

The current study found that H19 has four regulatory functions (Schoenfelder et al., 2007). ① H19/miR-675 axis: H19 is the precursor for the miR-675 and the sequence of miR-675 is found in the first exon of the H19 sequence. H19/miR-675 axis has many roles in cell growth and proliferation (Keniry et al., 2012). For instance, the H19/miR-675 axis has been implicated in the growth and migration of human squamous cell carcinoma cells (Zhang et al., 2021a). ② Binding to miRNAs: H19 is known to bind a range of microRNAs, including miRNA-138, let-7 and miRNA-200a and separately buffer their individual effects on target genes, such as Vimentin, Integrin β3, and ZEB1/ZEB2 to change the phenotype of the diseases (Liang et al., 2015; Zhao et al., 2019a; He et al., 2019). ③ Recruiting proteins: H19 is also known to bind to a range of proteins, thereby modulating downstream activities. By this mechanism, H19 binds to EZH2 and inhibits E-cadherin expression, promoting the metastasis of bladder cancer cells (Luo et al., 2013; Zhu et al., 2018). ④ Epigenetic effects: epigenetic effects have also been reported for H19 that binding to gene promoter regions, histone modification enzymes and transcription factors to affect gene expression. For instance, H19 is thought to induce p-glycoprotein expression and by regulating the methylation of MDR1 promoter with the effect that drug resistance is promoted in hepatocellular carcinoma cells (Tsang and Kwok, 2007). The physiological effects outlined above confirm that H19 is a biomarker and potential therapeutic target in diseases such as cancers and cardiovascular diseases (Liu et al., 2019a). We have plotted the mechanism of H19 in Figure1.

## 4 The regulatory mechanisms of H19 in pulmonary diseases

Lung cancer, pulmonary arterial hypertension, asthma, and many other diseases are all regarded as systemic disorders with manifestations in the lung. Many such disorders have reported the involvement of H19, among which lung cancer makes frequent appearances in the medical literature. Different effects have been characterized in different cell types. For example, H19 promotes pulmonary fibrosis by inhibiting the expression of miR-140 in human lung fibroblasts (HFL), leading to enhanced deposition of extracellular matrix (Wang et al., 2019). Further specific regulatory roles have been recorded in airway smooth muscle cells (ASMC), myoblasts, cardiomyocytes, small cell lung cancer lines (SCLC), and non-small cell lung cancer lines (NSCLC). Pathways and downstream targets are summarized in Table 1. All the above lung diseases are worthy of further discussion during this review with respect to the roles of H19.

**TABLE 1:** The miRNA, pathways and proteins interacting with H19.

Diseases	Target	Downstream pathways or molecules	References	Physical characteristics
Asthma	PI3K/Akt	NF-Κb/Muc5ac	Chen et al. (2021a)	Airway remodeling
PAH	Let-7b	ACE-AngII-AT1R	Sun et al. (2019)	Right ventricular hypertrophy
	miR-675	E2F1/EZH2	Omura et al. (2020)	
	YB1	Col1a1	Choong et al. (2019)	Pulmonary vascular remodeling
IPF	miR-29b	FGFb1	Tang et al. (2016)	Phenotypic transformation disorder and extracellular matrix collagen deposition in alveolar epithelial cells
	miR-140	TGF-β/smad3	Wang et al. (2019)	
	miR-196a	Col1a1	Lu et al. (2018)	
Lung injury	miR-181a	Runx2	Wu et al. (2018)	Inflammation
	miR-29b-3p	HGMB1/TLR4	Tan et al. (2021)	
	miR-17	STAT3	Zhang et al. (2020)	
Pneumonia	miR-22-3p	NLRP3	Sun et al. (2021)	Congestion, Interstitial inflammation and diffuse alveolar damage
	miR-140-5p	TLR4	Yang (2022)	Metastasis and diffusion
SCLC	miR-140-5p	FGF9	Li et al. (2020)	
NSCLC	miR-484	JNK	Zhang et al. (2021)	Proliferation and metastasis
	CDH1 promoter	DNMT1/DNMT3A	Gao et al. (2019)	
	miR-29b-3p	STAT3	Liu et al. (2019)	
	miR-148b-3p	DDAH1	Huang et al. (2020b)	
	microRNA-107	NF1	Qian et al. (2018)	
	EZH2	PTEN	Xu et al. (2019)	
	miR-19b-3p	FTH1	Zhang et al. (2022)	

### 4.1 Asthma

Asthma is characterized by episodic and reversible airway constriction as part of an inflammatory response to environmental allergens, infections and irritants. It is a complex, multifactorial immune-mediated process with multiple clinical types and airway remodeling, promoted by abnormal proliferation and migration of smooth muscle cells (Lambrecht and Hammad, 2012; Patel and Teach, 2019). The work of Chen has shown that H19 regulated the expression of mucoprotein 5AC (Muc5ac) through the PI3K/Akt/NF-κB pathway in an *in vitro* mouse model of inflammation in which the expression of H19 was low (Chen et al., 2021a). And Low expression of H19 has been proposed as a protective mechanism in mouse models of asthma. The inhibitors of phosphatidylinositol 3-kinase (PI3K) eliminated the promotion of Muc5ac expression by H19 through dephosphorylation of protein kinase B (Akt) and subsequent activation of nuclear factor kappa-B (NF-κB). Muc5ac is a central effector of allergic inflammation involved in airway hyper reactivity (AHR) (Evans et al., 2015). The activity of Muc5ac may lead to mucus blockage and airway inflammation worsening asthma symptoms. Therefore, H19 inhibition may alleviates the development of asthma by downregulating Muc5ac.

### 4.2 Pulmonary arterial hypertension

Changes in pulmonary vascular structure or function may lead to increased vascular resistance and arterial pressure culminating in the pathological presentation of pulmonary arterial hypertension (PAH). The condition is defined as a resting mean pulmonary arterial pressure (mPAP), measured by right heart catheterization, in excess of 25 mmHg (Galie et al., 2016). Pulmonary vascular remodeling and right ventricular (RV) hypertrophy or even failure may result from PAH (Hoffmann et al., 2016).

Vascular remodeling results from excessive proliferation of pulmonary artery smooth muscle cells and the dysfunction of arterial endothelial cells (Kim, 2014). There is a great deal of evidence to suggest the involvement of the H19-let-7b axis in vascular remodeling (Sun et al., 2019). H19, in a role as a competing endogenous RNA (ceRNA), binds to let-7b to down-regulate its expression and upregulate the expression of cyclinD1. The result is the proliferation of vascular smooth muscle and vascular remodeling (Sun et al., 2019). It has also been shown that by binding and sequestration of let-7b, H19 can upregulate the expression of the angiotensin I receptor-1 (AT1R) in rat pulmonary artery smooth muscle cells (PASMCs) stimulated by platelet-derived growth factor-BB (PDGF-BB) (Su et al., 2018). The binding of angiotensin II to AT 1 R activates the MAPK signaling pathway to promote the proliferation of blood vessels and smooth muscle cells, contributing to vascular remodeling.

The foregoing results all indicate a pathological role for H19 in PAH. However, there is a contradictory finding. Wang reported elevated H19 in a melatonin-mediated rat model of PAH, in which the H19-miR-200a-PDCD4 axis played a therapeutic role (Wang et al., 2018a). There may be environmental and time exposure factors accounting for the opposite finding.

H19 regulated cardiomyocyte (CM) hypertrophy, promoted CM apoptosis, and stimulated proliferation and fibrosis of cardiac fibroblasts *in vitro*, all of which are related to the progression of right ventricular failure (Liu et al., 2018; Choong et al., 2019). Omura used CMs from rats with pulmonary hypertension to demonstrate that H19 silencing upregulates the histone methyltransferases, enhancer of Zeste homolog 2 (EZH2) and E2F1 (Omura et al., 2020). Upregulation of EZH2 may contribute to improving CM cross-sectional area. However, any specific regulatory relationships among H19, EZH2, and E2F1 remain unclear. E2F1 is known to be a target of miR-675 and a trans-activator of EZH2 (Ma et al., 2018). Therefore, H19 is likely to inhibit the expression of EZH2 through the miR-675-E2F1-EZH2 axis. EZH2 exerts epigenetic effects to inhibit the expression of sine oculis homeobox homolog 1 (six1) (Gao et al., 2020). Decreased six1 reduced the expression of cardiac sarco-endoplasmic reticulum Ca2+ ATPase marker (SERCA2a) (Meyer et al., 1995). All in all, H19 knockdown may reduce myocardial hypertrophy and heart failure through this cascade.

In addition, H19 inhibits the expression of y-box binding protein 1 (YB1), leading to de-inhibition of type 1 collagenα1 (Col1α1) and a variety of miRNAs, such as miR-29b. The resulting changes in the extracellular matrix may reduce CM survival and promote fibrosis (Choong et al., 2019).

In conclusion, we suggest that H19 may have a central role in the process of vascular smooth muscle cell proliferation and CM extracellular matrix alteration making it a suitable drug target for PAH.

### 4.3 Idiopathic pulmonary fibrosis

Idiopathic pulmonary fibrosis (IPF) has a high mortality rate, and there is no effective drug treatment (Nathan and Meyer, 2014). Two features characterize IPF: the phenotypic transformation of alveolar epithelial cells and excessive deposition of extracellular matrix collagen (Ley et al., 2011). The involvement of miRNAs has been confirmed in pulmonary fibrosis, and H19 has been shown to bind the 3′UTR and inhibit the expression of the fibrosis regulator, miR-29b (Tang et al., 2016). Moreover, miR-29b inhibits col1a1 *via* an effect on the PI3K/Akt pathway (Li et al., 2012). Transforming growth factor1 (TGFb1) induces deposition of extracellular collagen to mediate pulmonary fibrosis *in vitro*. Its role in cell proliferation requires the activity of miR-29, and miR-29 overexpression inhibits TGFb1-induced lung fibroblast proliferation (Tang et al., 2016). Indeed, miR-29 may regulate fibrosis by regulating the TGFb1 signaling pathway (Li et al., 2016). Besides, Lu also confirmed that H19 could promote IPF. As a ceRNA, H19 can compete with miR-196a to positively regulate colla1 and promote the development of IPF (Lu et al., 2018). Similarly, Wang has reported that H19 exerts a promotional effect on pulmonary fibrosis by reducing the expression of miR-140, inhibiting the TGF-b/smad3 pathway (Wang et al., 2019). Optimal levels of TGF-β-smad3 signaling appear to be critical for secondary alveolar septum formation. Over-expression of TGF-β and TGF-β3 receptors may lead to invasive and transient pulmonary fibrosis, respectively (Warburton et al., 2013).

### 4.4 Lung injury

There are many types of lung injury, and acute lung injury (ALI) is the most common one. Inflammation is the main feature of lung injury (Vishnupriya et al., 2020). H19 has been confirmed to be closely associated with various inflammatory genes, regulating the expression of inflammatory genes (Wang et al., 2020a). Wu’s study showed that H19 was significantly expressed in LPS-induced acute lung injury in rats, and overexpression of H19 may be a protective mechanism (Wu et al., 2018). In contrast to the role of H19 in promoting disease progression in most diseases, overexpression of H19 negatively regulated miR-181a, promoting the expression of runt-related transcription factor 2 (Runx2), alleviating LPS-induced cell damage, and ameliorating LPS-induced acute lung injury (Wu et al., 2018). The same experiment confirmed that Runx2 activated the JNK and Notch pathways to promote cell proliferation and survival. In this study, H19 was shown to promote the proliferation of lung fibroblast cells (MRC-5 cells) to attenuate LPS-induced lung injury. However, in IPF, the overexpression of H19 promotes the proliferation of fibroblasts, which will cause the aggravation of IPF. For different diseases, the promotion of lung fibroblast cells proliferation by H19 has the opposite effect on the development of disease.

The incidence of smoke-induced lung injury (SILI) involves inflammatory stimulation (Guo et al., 2019). Ginsenoside Rb3 has been shown to inhibit H19 and play a therapeutic role in SILI. H19 acts as a sponge for miR-29b-3p to regulate its expression negatively. In addition, miR-29b-3p inhibited the expression of toll-like receptor 4 (TLR4) and alleviated lung injury by inhibiting the expression of HMGB1 mRNA (Tan et al., 2021). TLR4 promoted the production of inflammatory mediators (Yang et al., 2020).

Another lung injury associated with inflammation is bronchopulmonary dysplasia (BPD), which is disproportionately common in infants (Gilfillan et al., 2021). Zhang proved that inhibition of H19 could significantly improve alveolar congestion and inflammatory infiltration in BPD mice (Zhang et al., 2020b). H19 has a negative regulatory effect on miR-17, competitively binding to it and upregulating STAT3 to promote inflammation. STAT3 activates inflammatory pathways, including NF-κB in cancer (Yu et al., 2009). Therefore, regulation of the H19/miR-17/STAT3 axis may have therapeutic effects on BPD.

### 4.5 Pneumonia

Pneumonia is an acute respiratory tract, including community-acquired pneumonia (CAP) and hospital-acquired pneumonia (Torres et al., 2021). Infection of the lungs and damage to the immune barrier by pathogens are the leading cause of pneumonia (Torres et al., 2021). Sun showed that H19 was significantly increased in LPS-induced pneumonia models and sponging of miR-22-3p positively regulated NLRP3 to promote cell pyroptosis (Sun et al., 2021). The NLRP3 inflammasome promoted the activation and release of protease caspase-1 to induce gasdermin D-mediated pyroptosis (Huang et al., 2021). Another study also showed that inhibiting the expression of H19 can alleviate inflammation by regulating miR-140-5p/TLR4 axis in pneumonia (Yang, 2022). The LPS receptor, TLR4, may promote LPS-mediated endogenic transduction of inflammatory signals (Zamyatina and Heine, 2020). In conclusion, H19 plays a positive promoting role in the pneumonia model. Therefore, H19-targeted therapy may play a role in the treatment of pneumonia.

### 4.6 Lung cancer

Lung cancer is the most common cancer-type worldwide (Sung et al., 2021). It can broadly be divided into two types: non-small cell lung cancer (NSCLC) which accounts for 80%–85% of total lung cancer (40% adenocarcinoma, 25%–30% squamous cell carcinoma, and 10%–15% large cell carcinoma) and small cell lung cancer (SCLC) (Schabath and Cote, 2019). H19 may promote the proliferation and migration of tumor cells.

#### 4.6.1 Small cell lung cancer

SCLC only accounts for about 15%–20% of lung cancer cases. However, its propensity for metastasis means that about two-thirds of patients have distant metastatic disease at the initial diagnosis, giving them an abysmal prognosis and low survival rate (Hou et al., 2012). Knockout of H19 has been shown to inhibit the proliferation and migration of SCLC cells. Li demonstrated that H19 regulates the expression of the tumor suppressor gene, miR-140-5p (Li et al., 2020b). The expression of miR-140-5p had an inhibitory action on cell invasion and epithelial-mesenchymal transformation of oral squamous cell carcinoma (Zhao et al., 2019b). The oncogene, fibroblast growth factor (FGF9), is a target of miR-140-5p in SCLC. And miR-140-5p can bind to 3′-UTR of FGF9 and inhibits its expression (Wang et al., 2020b). Therefore, through negative regulation of miR-140-5p, H19 stimulates the tumor-promoting activity of FGF9 in SCLC. The detailed findings expose the possibility that H19 could be a therapeutic target in SCLC and have potential as a biomarker. Additional mechanistic details should be explored.

#### 4.6.2 Non-small cell lung cancer

NSCLC includes adenocarcinoma, squamous cell carcinoma, and large cell carcinoma, among which lung adenocarcinoma accounts for the highest proportion. H19 has been shown to reduce miR-484 expression in A549 cells *in vitro* (Zhang et al., 2021b). Through its negative regulation of the cytokinesis and proliferation factor, Rho-associated kinase 2 (ROCK2), miR-484 would usually exert a tumor-suppressing activity (Hartmann et al., 2015; Liu and Li, 2020). ROCK2 overexpression has been shown to increase the levels of phosphorylated stress-activated protein kinase (JNK), transcription regulation factors C-Jun, and mesenchymal markers but to decrease levels of epithelial markers. This suggests that ROCK2 regulates the epithelial-mesenchymal transformation (EMT), including loss of cell polarity and increased mobility (Zhang et al., 2021b). EMT promotes invasive metastasis, resistance to anti-tumor drugs, and various stress responses in tumor cells (Nieto, 2017). Therefore, H19 knockout influences proliferation, invasion, and metastasis by upregulating mir-484 and down-regulating ROCK2 to inhibit the EMT in lung cancer cells.

It has been suggested that the activity of H19-induced DNA methyltransferase1 (DNMT1) and DNA methyltransferase3 (DNMT3) promoted methylation and silencing of the Cadherin 1 (CDH1) promoter (Gao et al., 2019). In addition, CDH1-inhibition may promote TGF-β1-induced EMT and tumor invasion in breast cancer (Cano-Gonzalez and Lopez-Rivas, 2016). These findings suggest that inhibition of H19 and CDH1 methylation may inhibit EMT and cell proliferation, promoting apoptosis of lung adenocarcinoma cells. Moreover, H19, as a ceRNA, targets miR-29b-3p to limit its expression (Liu et al., 2019b). The target gene of miR-29b-3p, signal transducer and activator of transcription 3 (STAT3), promotes differentiation of bone-marrow-derived inhibitory cells (MDSCs) and acts as a tumor promoter (Liu et al., 2019b). It may be that STAT3, activated by H19, increases proliferation and decreases apoptosis of lung adenocarcinoma cells and activates the expression of EMT-specific proteins.

H19 has been shown to regulate miR-148b-3p and promote the expression of dimethylarginine dimethylaminohydrolase 1 (DDAH1) through negative feedback in lung adenocarcinoma cells resistant to gefitinib (Huang et al., 2020a). DDAH1 has been shown to inhibit the EMT in gastric cancer, suppressing tumor growth, but is considered a risk factor in breast cancer and cardiovascular systems (Palm et al., 2007; Cano-Gonzalez and Lopez-Rivas, 2016; Hulin et al., 2017). Further research is necessary to explain why DDAH1 shows adverse effects in different disease systems. In addition, H19 may act as a competitive inhibitor of microRNA-107, which usually modulates degradation of the RAS signal transduction pathway negative regulator, NF1 (Qian et al., 2018). Proliferation and migration of NSCLC cells would thus increase in the presence of H19. NF1 is known to regulate metastasis of hepatocellular carcinoma cells (Li et al., 2015). And NF1 is a GTP-activating protein that acts as a Ras protein shutdown signal. Patients who lack NF1 have higher levels of RAS-GTP, which promotes cancer cell growth and differentiation (Brundage et al., 2014). The forkhead box protein F2 (FOXF2) transcription factor has been shown to stimulate H19 transcription, leading to H19-mediated silencing of the tumor suppressor gene, PTEN, by recruitment of EZH2 (Xu et al., 2019). Thus, the progression of NSCLC is exacerbated. Another study showed that Curcumenol could effectively inhibit the expression of H19 and promote the occurrence of ferroptosis in lung cancer (Zhang et al., 2022). H19 acts as the “ceRNA” of miR-19b-3p and promotes the expression of FTH1, which plays a promoting role in lung cancer. FTH1 is an inhibitor of ferroptosis (Tian et al., 2020). Therefore, Curcumenol can target H19 to treat lung cancer.

**FIGURE 2:**
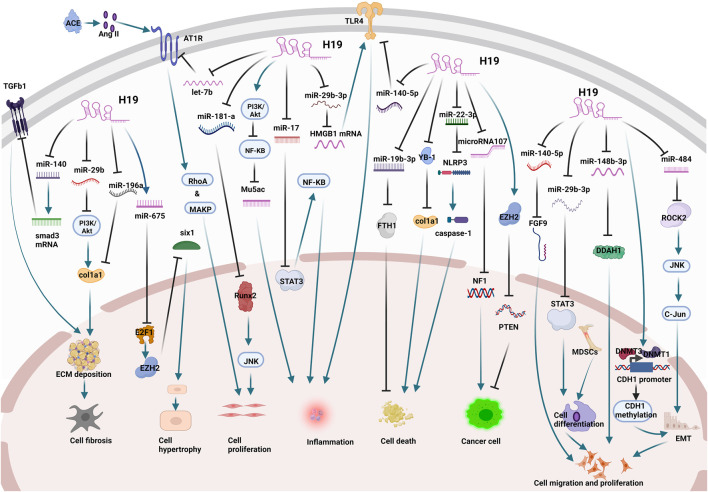
The mechanisms and related targets of H19 in lung diseases. H19 acts as a sponge for miR-140, miR-29b, and miR-196a to promote cellular fibrosis. H19 promotes cell hypertrophy by splicing to produce miR-675. H19 acts as a sponge for miR-21 and miR-181a to promote cell proliferation. H19 promotes inflammation by acting as a sponge for miR-17, miR-29b-3p, and miR-140-5p and regulating the PI3K/Akt pathway through epigenetic pathways. H19 regulates cell death by binding to YB1 and acting as a sponge for miR-22-3p and miR-19b-3p. H19 promotes cell carcinogenesis by acting as a sponge for microRNA107 and regulating EZH2 expression through epigenetic pathways. H19 acts as a miR-29b-3p, miR-148b-3p, and miR-484 sponge and regulates the expression of CDH1 promoter through epigenetic pathways to promote cancer cell proliferation and metastasis.

Finally, we summarized the current studies on H19 in lung diseases (Figure 2) to explain the potential therapeutic value of H19 in lung diseases.

## 5 The drugs targeting H19

A summary of the drugs targeting H19 from a search of previous publications is shown in Table 2. It is not difficult to see from the table that there are currently three drugs targeting H19 to treat lung diseases, namely melatonin, Curcumenol and Ginsenoside Rb3. Both Curcumenol and Ginsenoside Rb3 inhibit H19 expression through the H19/miR-19b-3p/FTH1 axis and the H19/miR-29b-3p/HGMB1/TLR4 axis to promote ferroptosis in lung cancer cells and reduce inflammation (Tan et al., 2021; Zhang et al., 2022). But Wang showed that melatonin upregulated H19 expression to suppress PAH, in contrast to other reports that H19 promoted this disease (Wang et al., 2018a). In this study, H19 was identified as a positive factor that inhibits the development of the disease.

**TABLE 2:** The drugs targeting H19.

Drugs	Diseases	Year	References	Biological mechanisms
Ginsenoside Rb3	Smoke-induced lung injury	2021	Tan et al. (2021)	Inhibiting the expression of H19, HMGB1, and TLR4, promoting the expression of miR-29b-3p. And then alleviating smoke‐induced lung injury
Curcumenol	Lung cancer	2022	Zhang et al. (2022)	Inhibiting the expression of H19 and FTH1, promoting ferroptosis and the expression of miR-19b-3p
Dihydroartemisinin	Liver fibrosis	2021	Xia et al. (2021)	Inhibiting H19 transcription and reducing signaling by H19-AMPK, thereby preventing liver fibrosis
Metformin	Cerebral ischemia-reperfusion	2019	Zeng et al. (2019)	Inhibiting the expression of H19 can promote the expression of miR-148a-3p, thus decreasing the expression of Rock2 to inhibit oxidative stress response
	Polycystic ovary syndrome	2019	Chen et al. (2019)	Increasing the expression of miR-29b-3p by inhibiting H19, thus inhibiting the expression of MMP-9 and MMP-2
	Gastric cancer	2019	Li et al. (2019b)	Decreasing the expression of H19, thereby activating AMPKα and inhibiting MMP9
	Pre-eclampsia	2019	Shu et al. (2019)	Reducing H19, promoting the expression of miR-148a-5p and miR-216-3p, and then decreasing the expression of P28 and EBI3 proteins
	Diabetic nephropathy	2020	Xu et al. (2020)	Decreasing the expression of H19 and TGF-β1, promoting the expression of miR-143-3p, and reducing cell proliferation, inflammation, and ECM accumulation
Melatonin	Ischemic heart diseases	2016	Cai et al. (2016)	By promoting the expression of miR-675, the senescence of cardiac grandmother cells was inhibited
	Early brain injury following subarachnoid hemorrhage	2018	Yang et al. (2018)	Promoting the expression of H19, miR-675-3p, and NGF, inhibiting the expression of P53 and LET-7A, and then inhibiting apoptosis
	Pulmonary Hypertension	2018	Wang et al. (2018a)	Upregulating H19, miR-675-3p, and PDCD4, downregulating miR-200a and IGFR1, and then reducing vascular remodeling and PAH.
Atorvastatin	Acute myocardial infarction	2020	Huang et al. (2020b)	H19 in exosomes and its downstream signaling pathway mediate blood vessels to protect the heart
Levonorgestrel	Adenomyosis	2020	Liang et al. (2020)	Increasing the expression of H19 and decreasing miR-17 and TLR4 to promote apoptosis and inhibit inflammation
Valproic acid	Ovarian cancer	2018	Sajadpoor et al. (2018)	Negatively regulating the expression of H19 and EZH2, inducing apoptosis and inhibiting proliferation of cancer cells
5-Azacytidine	Rhabdomyosarcoma	2015	Tarnowski et al. (2015)	Activating H19 and miR-675 by demethylation of DMR at IGF2-H19 inhibits rhabdomyosarcoma cell proliferation
Geniposide	Hypoxic-ischemic encephalopathy	2019	Yuan and Zheng, (2019)	Promoting the expression of H19, activating the PI3K/AKT and Wnt/β -catenin pathways, and inhibiting cell apoptosis
Astragaloside IV	Atherosclerosis	2019	Song et al. (2019)	Promoting H19 expression, inhibiting DUSP5, then attenuating autophagy and mineralization of VSMCs in atherosclerosis
6-Gingerol	Myocardial ischemia/reperfusion injury	2021	Lv et al. (2021)	Increasing the expression of H19 and ATG7, inhibiting the expression of miR-143, and promoting autophagy to alleviate myocardial injury
Huaier Extract	Breast Cancer	2017	Wang et al. (2017)	Inhibiting the expression of H19 and miR-675, promoting the expression of CBL, inhibiting the proliferation of breast cancer cells, and inducing apoptosis
Cinnamaldehyde	Inflammatory bowel disease	2021	Qu et al. (2021)	Inhibiting Th17 cell differentiation by the S1P2 pathway and inducing inflammation by regulating H19 and MIAT.
Berberine	Nonalcoholic fatty liver disease	2021	Wang et al. (2021b)	Inhibiting the expression of H19 can alleviate liver fibrosis
Icariin	Aberrant proliferation of retinal pigment epithelial	2020	Zhang et al. (2020c)	Promoting the expression of H19, p53, and p21, inhibiting cell proliferation

Currently, we have not found drugs that can target H19 to treat diseases such as pulmonary fibrosis, asthma and pneumonia. Drugs targeting H19 remain a potentially fruitful area of research.

The pathogenesis of asthma and pneumonia are all related to the production of inflammation, and pulmonary fibrosis is related to the production of fibrosis, and H19 is highly expressed in these diseases without exception. Studies by others have found that metformin suppressed inflammation in diabetic nephropathy by inhibiting H19 expression and that dihydroartemisinin inhibited the development of liver fibrosis (Xu et al., 2020; Xia et al., 2021). Therefore, we can also hypothesize whether drugs such as metformin and dihydroartemisinin may target H19 in lung disease. We prospect that future research may demonstrate the applicability of these drugs.

In recent years, monomeric chemical constituents in botanicals have been widely used to target H19 to inhibit disease progression. Using existing online databases, including TCMSP and miRbase, facilitates the bioinformatics-based search for drugs targeting H19.

## 6 The molecular therapy targeting H19

RNA-based therapeutics, including various antisense oligonucleotides (ASOs), small interfering RNAs (siRNAs), and miRNA sponges, have been approved by the European Medicines Agency (EMA), demonstrating the clinical feasibility of RNA therapeutics (Winkle et al., 2021). However, no effective lncRNA-based therapy has been approved in the clinic, and most are in Phase II or Phase III clinical development. The diverse functions of lncRNAs indicate various opportunities for their therapeutic targeting, and corresponding molecular drugs can be developed by adjusting lncRNA modes of action, such as transcriptional inhibition, post-transcriptional inhibition, or interaction with proteins. We summarize possible future molecular therapies targeting H19.

### 6.1 The therapies based on modification

#### 6.1.1 The small molecule inhibitors of H19

With the continuous discovery of lncRNA structural information and its functional motifs, the design or discovery of small molecule inhibitors of related targets has become a reality. For example, the methylation inhibitor 5-azathioprine-2′-deoxycytidine (5-aza-dC) inhibited the methylation of the ICR region of the H19 gene and down-regulated the expression of the H19 gene (Zhao et al., 2012).

#### 6.1.2 The constructs of H19 regulatory sequences

The regulatory sequence of H19 has been evaluated as a promising and safe targeted therapy in phase 1 and 2 studies in pancreatic cancer (Hanna et al., 2012). Hochberg et al. constructed a vector named DTA-H19, and it was regulated by the regulatory sequences of H19. The vector can selectively kill H19-positive cells by expressing the diphtheria toxin A chain gene. Anticancer effects have been demonstrated for this treatment in phase I/IIa clinical studies in patients with superficial bladder cancer, unresectable pancreatic cancer, or ovarian cancer (Sidi et al., 2008; Hanna et al., 2012; Gofrit et al., 2014; Lavie et al., 2017). Furtherly, another new DTA vector regulated by double regulatory sequences, H19-DTA-P4-DTA, was subsequently developed, with better anticancer effects than before (Amit and Hochberg, 2010).

#### 6.1.3 The RNA interfering drugs of H19

The developments of nucleic acid drugs siRNA and antisense oligonucleotides (ASOs) make the development of drugs targeting interfering lncRNAs possible. The development of siRNAs and antisense oligonucleotides (ASOs) have the same effect on degrading the lncRNAs, having pointed out another direction to knock down the expression of lncRNAs (Chen et al., 2021b). For instance, The researchers found that the expression of lncRNA DANCR could be modulated with siRNA nanoparticles for the treatment of non-small cell lung cancer (Nicolescu et al., 2022). Other researchers have constructed an ASO targeting lncRNA MALAT1, which can significantly inhibit the metastasis and invasion of rat lung cancer cells. These third-generation modified ASOs use Au nanoparticles as a carrier, which maintain the stability and good biological activity of ASOs (Gong et al., 2019).

### 6.2 The therapies based on delivery mode

Effective delivery methods may solve the clinical translation of RNA therapy, so we list promising delivery methods for H19-targeting drugs in the future.

#### 6.2.1 The endogenous carrier-exosome delivery

Cell-derived exosomes are a good delivery vehicle with minimal antigenicity and cytotoxicity, which achieve cell-to-cell information transfer (Bayraktar et al., 2017). It has been reported that H19 mediated the development of cancer cells such as hepatocellular carcinoma by means of exosomes (Conigliaro et al., 2015). Higher levels of H19 were also found in serum exosomes from bladder cancer (Wang et al., 2018b). The above suggests that delivering H19 through the exosome pathway may be a better drug delivery method in future studies.

#### 6.2.2 The exogenous carrier delivery

Currently, lipid nanoparticles (LNPs) are regarded as the most effective way to deliver siRNA by exogenous carriers due to their easy modification, high biodegradability, and high biocompatibility (Winkle et al., 2021). Ligand-conjugated siRNA therapy based on N-acetylgalactosamine (GalNAc) has shown promising results in hepatocytes (Rajeev et al., 2015). Another delivery mode of metal-based NPs has also attracted attention and may be combined with various compounds for delivery (Khoshnevisan et al., 2018). Therefore, these delivery methods are already undergoing clinical studies and are expected to be used for H19-targeted therapy in the future.

## 7 Conclusion and prospects

H19 has been extensively studied in cancer, endocrine, and cardiovascular diseases. Given that H19 plays an important regulatory role in lung diseases such as lung cancer, pulmonary fibrosis, and pneumonia, targeting H19 is a promising area of research. Of course, there are still some difficulties in solving the clinical translation of many RNA-based therapies, such as specificity and delivery of molecular drugs. But new chemical modification methods and delivery methods are constantly improving. In addition, drugs targeting H19 with plant-based, low toxicity and few side effects are gotten attention in recent years. Using existing botanical databases and combining with bioinformatics technology, active ingredients targeting H19 can be quickly found to facilitate subsequent research. We prospect that the molecular therapy targeting H19 and related molecular drugs will be applied to clinical therapies.
